# Editorial: Straight from the heart: Novel insights and future perspectives for cardiac repair

**DOI:** 10.3389/fcvm.2023.1149626

**Published:** 2023-02-14

**Authors:** Sveva Bollini, Monika M. Gladka, Anke M. Smits

**Affiliations:** ^1^Department of Experimental Medicine (DIMES), University of Genova, IRCCS Ospedale Policlinico San Martino, Genova, Italy; ^2^Department of Medical Biology, Amsterdam Cardiovascular Sciences, Amsterdam University Medical Centers, Amsterdam, Netherlands; ^3^Department of Cell and Chemical Biology, Leiden University Medical Center, Leiden, Netherlands

**Keywords:** cardiac repair, myocardial infarction, regeneration, therapeutic approaches, ischemic heart disease

As Editors of this Research Topic, we reviewed with great interest several articles and reviews on the relevant strategies for cardiac repair and discussed their translational potential. In this editorial, we will summarize the key aspects and main findings of the accepted articles.

In the last 20 years, cell therapies have initially ignited high expectations as novel strategies to address myocardial regeneration following injury and disease. While bona fide results in terms of the generation of new functional contractile cardiomyocytes using cell therapy have been broadly debated, several independent lines of investigation have reported beneficial effects following the infusion of progenitor cells on other aspects of cardiac disease. Here, Bassetti et al. discuss an updated overview of the impact of cell therapy on the treatment of patients affected by refractory angina (RA). Indeed, RA management denotes an unmet clinical need due to the disease increasing prevalence within the aging population. Nonetheless, progenitor cell-based therapies have been shown in preclinical studies and published trials to be safe and reduce angina symptoms while improving myocardial perfusion and supporting cardiac function in the long term. According to the authors, cell therapy may provide a reliable therapeutic strategy for RA patients as long as it is considered as a range of medicinal products to be developed according to specific regulatory pathways and international guidelines.

Recent progresses in cell biology and tissue engineering have shed new light on innovative strategies to optimize endogenous mechanisms of cardiac repair for ischemic heart disease; yet, cardiac regeneration still represents an extremely challenging goal for translational research. In this Research Topic, several different strategies have been proposed in order to provide cardiomyocyte reconstitution: bioengineering methods to obtain direct reprogramming of cardiac fibroblasts into induced and transdifferentiated cardiomyocytes (iCMs) by Paoletti and Chiono, and rebooting myocardial renewal *via* dedifferentiation, and proliferation of pre-existing cardiomyocytes, by Bongiovanni et al. In the first overview, microRNA (miRNA)-driven reprogramming of fibroblasts into iCMs is comprehensively discussed as a promising translational approach through local injection of the reprogramming molecules into myocardial fibrotic areas. Authors particularly focus on the relevance of biochemical and biophysical factors enhancing direct cardiac reprogramming and the definition of efficient and secure nanocarriers for miRNA delivery. Bongiovanni et al. instead focus their perspective on working strategies addressing direct stimulation of dedifferentiation and proliferation of pre-existing cardiomyocytes for heart regeneration. The authors describe how mammalian cardiomyocyte cell cycle activity is controlled during prenatal and postnatal age. They further discuss how fine-tuning of micro-environmental, extracellular and intrinsic molecular mechanisms affecting cell cycle checkpoints, cytoskeleton arrangement, and energetic metabolism can re-activate cardiomyocyte proliferative and extend their regenerative potential for future clinical translation.

The review by Lodrini and Goumans falls within a similar scope. However, instead of focusing on the formation of new cardiomyocytes, the authors discuss the cellular processes that ensue after MI, such as apoptosis, necrosis, autophagy, and cellular senescence. Intervening with these processes may result in cardioprotection and increased survival of functional cardiomyocytes after MI.

Also, mitochondrial metabolism has been shown to be crucial for homeostatic processes, including cell proliferation and differentiation and has profound implications during the development and regeneration of the heart. The regenerative capacity of the heart is lost by the first week after birth, partially due to a metabolic shift from glycolysis to fatty acid oxidation. In the review by Bae et al., the authors highlight the mechanisms that regulate cardiac metabolism and could be exploited for future intervention during development, disease, and regeneration.

The era of transcription profile analyses and transcriptional engineering at single-cell resolution is fast evolving. In the review by Schoger et al., the authors discuss how single-cell transcriptomics has extended our knowledge and opened the door for emerging CRISPR/Cas9 technologies in clinical applications. Single-cell transcriptomics can identify changes in the cellular composition of the heart and heterogeneity within the same cell types in healthy and diseased hearts. Integrating this information with the revolutionizing CRISPR/Cas9 technology will greatly advance medical research and open a new chapter of precision and personalized medicine. In the last few years, CRISPR-mediated gene editing has already entered the clinic, making the application of CRISPR/Cas9 approaches a realistic option for more specific treatments of different cardiac diseases.

Streef and Smits offer another perspective on endogenous repair mechanisms by focusing on a cardiac cell type with a specific role in heart development in the embryo: the epicardium. These epicardial cells contribute to formation of cardiac tissue during embryogenesis by contributing cells and by producing paracrine factors. In the adult heart, the epicardium is activated upon injury, but partcipation to cardiac tissue formation is limited. By summarizing data from recent cardiac single-cell studies, Streef and Smits provide insights into the cellular composition of the epicardium during cardiogenesis and in the adult heart including cross-species differences. This information could help optimize the post-MI response of endogenous cell types with a reparative potential.

One of the sequalae of MI is sudden cardiac death (SCD), which is often the result of arrhythmogenesis. There is a potential association between cardiac sympathetic hyperinnervation and SCD. However, the underlying mechanism for hyperinnervation is unclear. Ge et al. investigate how the superior cervical ganglion -which participates in the sympathetic innervation of the heart- and the adjacent carotid body are affected over time after MI. Interestingly, they show that neuronal remodeling occurs within the ganglion toward an adrenergic phenotype and larger neuronal size. This effect is potentially mediated by the carotid body. These data provide a direction for the potential mechanism underlying hyperinnervation of the heart after MI. The authors suggest that the next step is to investigate the functional implications of these findings.

Other strategies to repair the heart after MI include the application of cardio-supportive devices such as cardiac patches. Feng et al. have produced a reduced graphene oxide (rGO)/silk fibroin-modified nanofibrous biomaterial that they sutured onto the infarcted rat heart. These patches can likely improve cardiac systolic function and ventricular remodeling by directly regulating an anti-fibrotic effect in cardiac fibroblasts.

In recent years, growing evidence indicates that non-coding RNAs play essential roles in regulating tissue homeostasis and pathophysiological conditions. Long non-coding RNAs (lncRNAs) are >200-nucleotide long transcripts that can interfere with gene expressions and signaling pathways in different tissues. LncRNAs have been found to be important in the field of cardiovascular medicine in both healthy and diseased conditions. Du et al. demonstrated that overexpression of the lncRNA N1LR improved cardiac function, reduced inflammatory response, and protected from cardiac fibrosis in a mouse model of acute myocardial infarction. This cardioprotective effect was due to decrease TGF-beta and Smad signaling, which activation is associated with cardiac fibrosis, making lncRNA N1LR a promising target for future clinical applications.

As this Research Topic has emphasized, the route to repair after a cardiac injury can be multifactorial, including approaches such as a reduction in damaged tissue (cardioprotection), the induction of cardiomyocyte proliferation, increasing the participation of local regenerative cell types, adjusting metabolism, hyperinnervation, or the fibrotic response. It has been a pleasure to edit this special issue and showcase the multitude of possibilities for cardiac repair that may hopefully contribute to a better quality of life for patients suffering from cardiac disease.

## Author contributions

SB, MG, and AS contributed to the concept or design of the editorial. All authors contributed to the article and approved the submitted version.

